# Supplementary material for: Time-series visual representations for sleep stages classification
Source: PLoS One. 2025 May 21;20(5):e0323689. doi: 10.1371/journal.pone.0323689 (PMC12094730; doi:10.1371/journal.pone.0323689)
Supplement: S1 Table — Accelerometer data consistently outperformed heart rate data in all scenarios, with the GAF achieving the highest balanced accuracy (82.36% ± 3.24%) when using patch ensembles. Patch-based ensembles significantly improved balanced accuracy compared to original images. (PDF) [file pone.0323689.s001.pdf]

		RP		GAF		MTF		Spectrograms	
Network	Config.	ACC	HR	ACC	HR	ACC	HR	ACC	HR
Eff.Net	Original	76.62	69.91	79.63	69.32	77.44	64.88	78.34	61.22
ACC + HR Ensembles	Simple Average	71.38		69.04		71.27		75.94	
	Weighted Average	76.54		77.57		74.95		78.19	
	Deep Features	75.59		77.61		75.43		77.12	
Eff.Net	Patch 1	76.80	66.36	76.79	65.69	74.30	63.21	72.04	51.07
	Patch 2	77.92	68.84	77.73	67.93	75.79	64.66	76.56	51.24
	Patch 3	73.49	64.18	73.17	64.74	71.45	61.17	71.66	51.25
	Patch 4	79.97	69.01	79.57	68.07	78.57	64.99	76.69	54.83
	Patch 5	76.85	66.06	78.70	69.04	76.82	65.12	76.43	52.83
	Patch 6	78.21	67.44	77.74	68.51	75.48	65.87	71.90	52.38
	Patch 7	74.06	62.86	72.37	62.61	72.38	60.77	77.72	53.14
	Patch 8	80.30	69.40	79.83	67.68	78.08	66.41	76.36	51.44
	Patch 9	76.32	64.99	77.74	63.94	74.79	63.21	71.94	53.07
Ensembles of Patches	Simple Average	80.39	71.39	82.36	73.71	80.03	70.21	79.11	54.90
	Weighted Average	80.38	71.28	82.04	72.15	80.20	68.78	79.01	54.91
	Simple Network	80.26	70.81	81.54	72.87	79.85	69.41	79.03	53.30
P. ACC + P. HR Ensembles	Simple Average	76.14		77.25		78.28		77.78	
	Weighted Average	79.28		81.44		80.32		78.64	
