# Supplementary material for: Time-series visual representations for sleep stages classification
Source: PLoS One. 2025 May 21;20(5):e0323689. doi: 10.1371/journal.pone.0323689 (PMC12094730; doi:10.1371/journal.pone.0323689)
Supplement: S2 Table — Heart rate data often outperformed accelerometer data in balanced accuracies (except for the Spectrogram), with the GAF achieving the highest balanced accuracy (62.18% ± 0.95%) when using patch ensemble. Patch-based ensembles significantly improved balanced accuracy compared to original images. (PDF) [file pone.0323689.s002.pdf]

		RP		GAF		MTF		Spectrograms	
**Network**	**Config.**	**ACC**	**HR**	**ACC**	**HR**	**ACC**	**HR**	**ACC**	**HR**
Eff.Net	Original	55.73	57.85	57.68	57.09	55.00	53.35	55.96	40.32
ACC + HR Ensembles	Simple Average	46.01		46.14		46.35		52.41	
	Weighted Average	50.24		50.10		50.37		<u>56.00</u>	
	Deep Features	<u>53.60</u>		<u>53.39</u>		<u>54.82</u>		51.01	
Eff.Net	Patch 1	53.68	54.08	54.96	53.93	51.58	51.48	52.72	35.33
	Patch 2	55.29	55.51	56.22	55.60	53.64	51.65	52.12	35.34
	Patch 3	44.11	45.85	52.06	51.47	45.79	47.92	43.61	35.63
	Patch 4	56.90	55.28	55.50	55.23	55.01	<u>52.79</u>	52.05	34.51
	Patch 5	54.62	54.36	56.18	54.72	53.54	50.62	52.04	<u>36.25</u>
	Patch 6	55.48	55.19	56.45	<u>55.91</u>	52.22	52.05	40.67	35.42
	Patch 7	49.71	50.06	51.24	50.28	46.45	48.03	<u>53.52</u>	35.34
	Patch 8	<u>57.44</u>	<u>56.04</u>	<u>57.88</u>	54.78	<u>55.32</u>	51.65	50.52	35.63
	Patch 9	54.21	53.93	55.88	53.25	52.54	50.92	48.29	35.33
Ensembles of Patches	Simple Average	59.19	<u>61.87</u>	<u>60.66</u>	<u>62.18</u>	<u>58.38</u>	<u>57.81</u>	55.53	<u>39.50</u>
	Weighted Average	<u>59.41</u>	61.46	60.16	61.57	58.30	57.34	<u>57.36</u>	39.28
	Simple Network	49.96	52.44	51.19	51.25	48.29	51.29	48.69	39.33
P. ACC + P. HR Ensembles	Simple Average	49.85		49.98		50.95		53.10	
	Weighted Average	<u>61.48</u>		<u>61.17</u>		<u>60.97</u>		<u>55.35</u>	
