# Supplementary material for: Time-series visual representations for sleep stages classification
Source: PLoS One. 2025 May 21;20(5):e0323689. doi: 10.1371/journal.pone.0323689 (PMC12094730; doi:10.1371/journal.pone.0323689)
Supplement: Attachment — Submitted filename: Response to Reviewers.pdf [file pone.0323689.s003.pdf]

Paper title: Time-series visual representations for sleep stages classification
Journal: PLOS ONE

February 21th, 2025

Dear Editor and Reviewers,

We would like to thank the editor and reviewers for their comments and suggestions. They were valuable and certainly contributed to improving the quality of our manuscript. In this revised version, we addressed all points raised by the reviewers and made adjustments to enhance clarity, methodology description, and data presentation.

The comments, along with our continued research during the review process, allowed us to refine and strengthen our manuscript. The revised version has been submitted through the online submission system, with the main changes highlighted.

We appreciate the time and effort dedicated by the reviewers and the editor and hope that this version meets the journal's criteria for publication. For any further questions, please do not hesitate to contact us.

Sincerely,

The authors

General Comment

After careful consideration, we feel that it has merit but does not fully meet PLOS ONE's publication criteria as it currently stands. Therefore, we invite you to submit a revised version of the manuscript that addresses the points raised during the review process.

Response: We thank the editor and the reviewers for diligently dealing with our submission and for the very constructive feedback.

In this new version, we addressed the points of both reviewers, and we modified the manuscript according to their insightful and accurate comments. We are grateful to both reviewers and the editor for their positive comments and for the time dedicated to the reviewing process. With our revised manuscript, we send a point-by-point response in which we did our best to address all of them.

***

Reviewer #1

The study explores the application of visual representations of time series data from smartwatches to classify sleep stages. The authors transform data collected from accelerometers and heart rate sensors into visual formats like Gramian Angular Fields (GAF), Recurrence Plots (RP), Markov Transition Fields (MTF), and spectrograms. These visual representations are processed using 2D convolutional neural networks (CNNs), with image patching and ensemble methods enhancing classification performance. The proposed method demonstrates superior accuracy compared to traditional raw data approaches. I have a few points that can help improve the manuscript.

1. EfficientNet is a class of models. The authors need to specify which specific EfficientNet model was used for the experiment.

Response: We agree with the reviewer's observation. To clarify, we now explicitly state that we used EfficientNet-B0 for our experiments. This information has been added to the "Materials and methods (Training and validation)" subsection.

***

2. The authors should clearly define the classification task they are working on. What exactly is the goal/benefit for different classification tasks, and what is the distribution of each class? Including a workflow or pipeline demonstration figure would greatly enhance clarity. For each task, the data distribution, including the actual number of cases (and their respective percentages), should be explicitly shown.

Response: Following the reviewer's suggestion, we have revised the manuscript to improve the clarity of our classification tasks. The "Introduction" section now explicitly describes the two classification tasks—two-stage (wake/sleep) and three-stage (wake/NREM/REM)—along with their practical relevance. The two-stage classification is useful for basic sleep detection, such as

in large-scale sleep monitoring or initial sleep disorder screenings, while the three-stage classification enables detailed analysis of sleep architecture, essential for clinical applications like diagnosing sleep disorders or assessing sleep quality.

In addition, we have included Table 1 in the “Materials and Methods (Dataset)” subsection. This table provides the number of samples and percentages per class, highlighting the dataset imbalance.

To further enhance clarity, we also added Figure 2, which illustrates the overall workflow of the proposed methodology, including the transformation of time series into visual representations and subsequent classification steps.

***

3. The authors mention a data imbalance issue. It would be helpful to know if they took any measures to address this problem or include it in a limitation discussion.

Response: To mitigate the impact of class imbalance, we applied class weighting, where the loss function assigns higher weights to minority classes, ensuring a more balanced contribution during training. The class weights w_c were calculated as the inverse of the class frequencies, normalized by the total number of samples:

$$w_c = \frac{N}{C \cdot n_C}$$

where N is the total number of samples, C is the number of classes, n_c is the number of samples in class c. For binary cross-entropy, used for sleep/wake classification, the weighted loss L for a binary classification problem is computed as:

$$L = -\frac{1}{N}\sum_{i=1}^{N}\left[w_{pos} \cdot y_i \cdot log(p_i) + w_{neg} \cdot (1 - y_i) \cdot log(1 - p_i)\right]$$

where y_i is the true label (0 or 1) for sample i, p_i is the predicted probability for the positive class, w_{pos} and w_{neg} are the weights for the positive and negative classes, respectively.

For categorical cross-entropy, used for sleep stages classification (three classes), the weighted loss L for a multi-class classification problem is computed as:

$$L = -\frac{1}{N}\sum_{i=1}^{N}\sum_{c=1}^{C} w_c \cdot y_{i,c} \cdot log(p_{i,c})$$

where $y_{i,c}$ is a binary indicator (0 or 1) for whether class c is the correct classification for sample i, $p_{i,c}$ is the predicted probability for class c, w_c is the weight assigned to class c. To address this comment, this strategy is now explicitly described in the “Materials and methods (Training and validation)” subsection.

***

4. For cross-validation, it is unclear whether the authors used a stratified split based on the label of each sample. Clarifying this would be helpful. It is also unclear if the accuracy in the paper is the average accuracy of the 5-fold cross validation. If so, standard deviation should be included.

Response: Following the suggestion, we have clarified these points in the revised manuscript. The “Materials and methods (Training and validation)” subsection now explicitly states that we used 5-fold cross-validation, ensuring that data from the same subject were never included in both training and validation within the same fold. Instead of a stratified split, we opted for a random split, allowing the model to capture the natural variability of sleep stage transitions, as now described in the “Materials and Methods (Training and Validation)” subsection.

Additionally, the “Materials and methods (Performance metrics and model evaluation)” subsection now specifies that the reported results correspond to the average balanced accuracy across the five folds, with the standard deviation included to quantify variability. Standard deviation values are reported in the “Results and discussion” section.

***

5. For better readability, I suggest placing the figure descriptions together with the figures.

Response: In the revised manuscript, figure descriptions have been placed together with their respective figures (figures 7-10 and figures 12-15).

***

6. The paper contains a large number of comparison results. The authors might consider highlighting the most significant ones and moving the rest to the supplementary material.

Response: We agree with the reviewer’s suggestion. To streamline the manuscript, we highlighted the most significant results in the main text and moved the full comparison tables (Tables 2 and 3) to the supplementary materials.

***

7. Conclusions and Discussion sections need more work. A summary of the study's goal should be included, along with how the findings support this goal. For example, why classifying sleeping stages is important and how can the method developed benefit research or improve human well-being.

Response: Following the reviewer’s suggestion, we revised the “Conclusion” section to better summarize the study's goal and clarify how the findings support it. The revised text now explicitly states that the objective was to enhance sleep stage classification using visual representations and deep learning. We also emphasize the importance of sleep stage classification for assessing sleep quality, detecting disorders, and advancing research.

***

Reviewer #2

The paper utilizes Gramian Angular Fields, Recurrence Plots, Markov models, Transition Fields, and spectrograms to process time series data collected from smartwatches, aiming to enhance the classification of sleep stages. The analyses and experiments are rigorous. However, the methods utilized are largely standard, and the paper lacks a degree of innovation.

Response: This study introduces a novel application for sleep stage classification using smartwatch data within a comprehensive full pipeline approach. While techniques such as Gramian Angular Fields (GAF), Recurrence Plots (RP), Markov Transition Fields (MTF) and spectrograms are established in other domains, their combined application and evaluation for this task represent a significant advancement. By assessing their effectiveness under identical conditions and comparing them to traditional approaches, this study bridges a critical gap that has not been addressed before in this context.

This study not only explores the application of these visual transformations but also in the integration of techniques such as image patching and ensemble methods, which significantly enhance classification performance. The combination of GAF with patching and ensemble techniques achieved a balanced accuracy of over 82% for two-stage classification (sleep/wake) and 62% for three-stage classification (wake/NREM/REM).

A direct comparison with other commonly used representations for sleep stage classification (feature extraction and direct use of raw data) highlights the advantages of visual representations, demonstrating improved classification accuracy and reinforcing their applicability in sleep monitoring, outperforming traditional methods by up to 8.9 percentage points in two-stage classification and 9.1 percentage points in three-stage classification. These results emphasize the ability of visual representations to capture fine-grained temporal structures and enhance prediction robustness.

Furthermore, this study provides novel insights into the distinct contributions of accelerometer and heart rate data. Accelerometer data proved more effective for distinguishing between sleep and wake states, while heart rate data played a critical role in three-stage classification. This finding underscores the complementary nature of these data sources and their importance for accurate sleep stage classification.

To address the reviewer's concern regarding innovation, we revised the “Introduction”, “Discussion”, and “Conclusion” sections to better emphasize the study's contributions. These revisions highlight how the proposed methodology advances the field by offering a non-invasive, cost-effective alternative to polysomnography and enabling more reliable health monitoring and early interventions. This study also paves the way for future research by demonstrating the potential of visual representations and deep learning techniques for sleep analysis.
