# Supplementary material for: Time-series visual representations for sleep stages classification
Source: PLoS One. 2025 May 21;20(5):e0323689. doi: 10.1371/journal.pone.0323689 (PMC12094730; doi:10.1371/journal.pone.0323689)
Supplement: Attachment — Submitted filename: Response_to_Reviewers_auresp_2.pdf [file pone.0323689.s004.pdf]

Paper title: Time-series visual representations for sleep stages classification
Journal: PLOS ONE

March 21th, 2025

Dear Editor and Reviewers,

We would like to thank the editor and reviewers for their constructive feedback and thoughtful suggestions. Their insights have been invaluable in improving the clarity and rigor of our manuscript.

In this revised version, we carefully addressed all the reviewers' comments. Specifically, we clarified methodological choices and provided additional justifications where necessary. We also analyzed the data split strategy in more detail to ensure the reliability of our results.

The revised manuscript has been submitted through the online submission system, with key changes highlighted. Below, we provide a point-by-point response to each reviewer's comments, detailing the revisions made.

We greatly appreciate the time and effort the reviewers and the editor invested in evaluating our work. We hope that this version meets the high publication standards of PLOS ONE. After this round of revisions, we believe our manuscript is suitable for publication.

Please feel free to reach out for any further clarifications.

Sincerely,

The authors

General Comment

After careful consideration, we feel that it has merit but does not fully meet PLOS ONE's publication criteria as it currently stands. Therefore, we invite you to submit a revised version of the manuscript that addresses the points raised during the review process.

Response: We thank the editor and the reviewers for diligently dealing with our submission and for the very constructive feedback.

In this new version, we addressed the points of both reviewers, and we modified the manuscript according to their comments. We are grateful to both the reviewers and the editor for their comments and the time they dedicated to the reviewing process. With our revised manuscript, we send a point-by-point response in which we did our best to address all of them.

***

Reviewer #1

The study explores the application of visual representations of time series data from smartwatches to classify sleep stages. The authors transform data collected from accelerometers and heart rate sensors into visual formats like Gramian Angular Fields (GAF),Recurrence Plots (RP), Markov Transition Fields (MTF), and spectrograms. The visual representations are then fed into a 2d neural network for classification. Most of the comments have been addressed.

On data split: Given the imbalanced distribution of the dataset (minority class only 7.7%), using random split instead of stratified split is not a good representation of the data distribution. Without stratification, a random split might lead to some folds having very few (or even no samples) from the minority class. Even with the weight adjustment on minority samples in training, this can still lead to unreliable performance estimates as the train and test set may not have the same distribution. I suggest replacing the results with stratified split or checking the class distribution for the random split to make sure the data distribution in each fold is reliably representing the overall distribution.

Response: We appreciate your insightful comment regarding the impact of using a random split instead of a stratified split in our cross-validation process. We acknowledge the concern that a random split may result in some folds having very few or no samples from the minority class, potentially affecting the reliability of performance estimates in particular scenarios.

To address this, we analyzed the class distribution across the five splits. We presented the results in the new Table 2 of the revised version ("Materials and methods / Training and validation" subsection). The table shows the percentage of Wake, NREM, and REM samples for each split in the training and validation sets. The training data maintains a stable class distribution across splits, ensuring a balanced representation during model learning.

While the validation distribution shows some variability—particularly in Split 1 and Split 2 for Wake and Split 5 for REM—this reflects real-world sleep data, where sleep stages are inherently imbalanced across different nights and individuals. Since the model is evaluated across multiple splits, the impact of these variations is minimized.

It is important to emphasize that our dataset consists of sleep time-series data, where each sample corresponds to data from a specific subject. To prevent data leakage, we ensured that data from the same subject were not simultaneously used for training and validation. Unlike traditional classification tasks where stratification can be applied at the sample level, our setup requires entire subjects to be assigned exclusively to either a training fold or a validation fold. Our decision to use a random split was also motivated by the goal of maintaining the natural variability of sleep stage transitions, which differ across nights and individuals.

Given the constraints imposed by subject-level separation, we believe that using a random split does not introduce substantial bias or compromise the reliability of our results in this study. Additionally, the data distribution in each fold reflects the overall distribution, enabling the model to be tested under conditions that closely resemble real sleep patterns.

While we appreciate the suggestion to replace the results with a stratified split, our analysis indicates that the observed stability in class distributions and the need to maintain subject independence make our current approach the most appropriate for this study.

We sincerely appreciate your valuable feedback. Please let us know if further clarification is needed.

***

Reviewer #2

All the review comments have been addressed, and the paper has been improved. Although I still believe the methods are standard and lack some innovation, the application and execution of these methods demonstrate creativity. There is potential for further development.

Response: We sincerely appreciate your time and effort in reviewing our paper. Your feedback has helped us refine our work, and we are grateful for your positive remarks regarding the application and execution of our methods. While we recognize that the techniques employed are well-established, we focused on demonstrating their effectiveness in a novel application. We agree that there is potential for further development, and we see this work as a foundation for future research.

Thank you again for your valuable insights and constructive evaluation.
